# Living as a family with a child on home mechanical ventilation and personal care assistants—A burdensome impact on family life

**DOI:** 10.1002/nop2.879

**Published:** 2021-05-05

**Authors:** Anette Bjerregaard Alrø, Charlotte Klitnæs, Charlotte Dahl Rossau, Pia Dreyer

**Affiliations:** ^1^ Department of Intensive Care Aarhus University Hospital Aarhus N Denmark; ^2^ Institute of Public Health Section of Nursing Aarhus University Aarhus C Denmark; ^3^ Bergen University Bergen Norway

**Keywords:** child, dependency, family nursing, home mechanical ventilation, long‐term illness, personal care assistants, qualitative research, respiratory treatment

## 1 BACKGROUND

Over the last few decades, long‐term life support technologies such as ventilators keep children with chronic respiratory insufficiency alive and have become a well‐established treatment in many Western countries (Carnevale et al., 2008). More respiratory‐ill children, for example, children with neuromuscular diseases, can get life‐sustaining treatment at home because of advances in neonatal and paediatric care, technological advances and development of suitable home equipment (Cancelinha et al., 2015). Children dependent on invasively or non‐invasively ventilation, receive the treatment in a home setting, called home mechanical ventilation (HMV), where personal care assistants (PCAs) provide surveillance (Lindahl & Kirk, 2019). The change in care from hospital to a home‐based setting reduces hospital admissions; however, it also requires advanced home care programme (Carnevale et al., 2006; Lewarski & Gay, 2007). Patient pathways with HMV are very demanding, complex and stressful for the families. Lewarski and Gay (2007) emphasize the need for an effective HMV‐management programme to support transition from hospital to home, creating a safe‐home environment, and training of PCAs and follow‐up.

Research indicates that having HMV not only affects the ventilator‐dependent child, but also the family, because the technology, its complexity and parenting responsibilities are overwhelming (Carnevale et al., 2008). Families, both children, well siblings and parents are all affected emotionally, physically and socially, and experience reduced quality of life (QOL) (Anderson & Davis, 2011; González et al., 2017; Mesman et al., 2013). *Emotionally*, parents have concerns about their child's physical, emotional and social well‐being (Carnevale et al., 2008), and they experience feelings of guilt, mood swings and fear caused by the daily threat of death (Flynn et al., 2013; Mesman et al., 2013). *Physically*, parents experience chronic fatigue and exhaustion (Carnevale et al., 2008). *Socially*, parents struggle with isolation and experience a range of having an impact of relationships with family and friends, and limited activities for the family (Flynn et al., 2013; Lindahl et al., 2011a; Mesman et al., 2013). Siblings to enduring‐ill children also experience their lives being affected and they can feel neglected, ignored, isolated and lonely in the family if their parents spend less time with them versus the ill child. This can result in jealousy, disgust and conflicts and sibling rivalry (Alderfer et al., 2010; Israelsson‐Skogsberg & Lindahl, 2017; Williams et al., 2009).

In a Scandinavian setting families are entitled to PCAs, both some hours during the day/night or continuously, which affects their family and everyday lives. The family home has to live up to hygiene and ergonomic requirements, which some families experience as resembling a public or hospital environment (Lindahl et al., 2011a). Having PCAs in one's home around the clock means limited privacy, and battles for control can occur (Lindahl & Kirk, 2019). It is important for PCAs and their families to develop a professional and stable relationship because they enter a long‐standing cooperation (Lindahl et al., 2011a). Families are dependent on help from PCAs, but on a different level due to the disease, however, PCAs bring vulnerability to families because they can come and go (Lindahl & Lindblad, 2013).

The financial cost for the families and social health care are also relevant matters, because pathways with HMV are very expensive for society and make up a great deal of the overall health expenses in most countries (González et al., 2017; Lindahl & Kirk, 2019).

Investigating the perspectives of parents and children on family life with HMV and PCAs is not only important for the families, but also for the social care and healthcare workers and health economics in society due to the huge expenses in this field.

## 2 AIM

To explore family life with a child on HMV and PCAs from a family perspective.

## 3 METHODS

### 3.1 Design

We undertook a qualitative study with semi‐structured interviews using a phenomenological‐hermeneutic approach influenced by Ricoeur (Dreyer & Pedersen, 2009). Following Ricoeur (Ricoeur, 1973a), interpreting a text means seeing something new in what is taken for granted and disclosing a new sort of being‐in‐the‐world. As such, the researchers in the study attempted to explain and understand the meaning of the interviews and promote a comprehensive understanding of the text, and thereby the study participants’ lived experiences (Dreyer & Pedersen, 2009). With inspiration from Ricoeur, we explored, interpreted and described the phenomenon “family life with HMV and PCAs.”

### 3.2 Data collection

Data were collected from March‐June 2019 consisting of interviews with 22 participants, divided in *n* = 5 children and *n* = 17 parents. The families are defined as a group consisting of one‐two parent(s) and their child/children living together as a unit.

The distribution of the interviews was as follows: Five interviews were conducted with five children aged 8–17 years, two of which were conducted without parents present and three with parents present. Seven interviews were conducted with the mother only and two interviews with the father only. In addition, four interviews where both mother and father were present and one as a family were conducted (Table 1). The remaining eight children in the included families did not participate due to different reasons; The little ones, which included four children had not developed a language, two other children had not developed language due to their illness and the last two ones had language difficulties due to their illness.

**TABLE 1:** The included participants and type of interview

	Child (gender)	Age	Respiratory assistance	PCAs (hours/day)	Family patterns	Participant and type of interview
***Family 1***	Boy	2 years	Non‐invasive HMV	24 hr./social and healthcare assistants and social and healthcare helpers (no help during daytime at weekends)	Living with mother, father and siblings	(1a) Mother (individual interview)
***Family 2***	Girl	8 years	Non‐invasive HMV	20 hr./nurses and social and healthcare assistants (no help from 16−20pm)	Living with mother and father	(2a) Mother (individual interview) (2b) Girl (individual interview)
***Family 3***	Girl	14 years	Non‐invasive HMV	24 hr./personal trained helpers	Living with mother, father and siblings	(3a‐b‐c) Mother, father and girl (group interview)
***Family 4***	Girl	13 months	Invasive HMV	24 hr./social and healthcare assistants	Living with mother and father	(4a‐b) Mother and father (couple interview)
***Family 5***	Boy	10 months	Non‐invasive HMV	24 hr./social and healthcare assistants	Living with mother and father	(5a‐b) Mother and father (couple interview)
***Family 6***	Boy	14 years	Non‐invasive HMV	8 hr. at night‐time/medical students incl. father on the team	Living with mother, father and siblings	(6a‐b) Mother and father (couple interview) (6c) Boy (individual interview)
***Family 7***	Boy	13 years	Non‐invasive HMV	11 hr. (night‐time)/social and healthcare assistants	Living with mother	(7a) Mother (individual interview)
***Family 8***	Boy	5 years	Invasive HMV	24 hr./nurses	Living with mother, father and siblings	(8a) Mother (individual interview)
***Family 9***	Boy	12 years	Non‐invasive HMV	0 hr./terminated PCAs (social and healthcare workers) 1 year ago	Living with mother, father and siblings	(9a) Mother (individual interview) (9b) Boy (individual interview)
***Family 10***	Boy	8 years	Invasive HMV	24 hr./social and healthcare assistants incl. mother	Living with mother and siblings	(10a) Mother (individual interview)
***Family 11***	Boy	4 years	Non‐invasive HMV	24 hr./nurses	Living with mother, father and siblings	(11a) Mother (individual interview)
***Family 12***	Girl	5 years	Invasive HMV	24 hr./ social and healthcare assistants	Living with mother, father and siblings	(12a) Father (individual interview)
***Family 13***	Boy	17 years	Non‐invasive HMV	24 hr./social and healthcare assistants and social and healthcare helpers incl. father	Living with father	(13a) Father (individual interview) (13b) Boy (individual interview)

Including children in different ages provided deeper understanding of their perspective. Each family decided whether they wanted an individual or a couple/family interview based on their wishes and preferences. A combination of types of interviews provided variation on family life with HMV and PCAs. The individual interviews provided a deeper understanding, whereas the couple/family interviews stimulated discussion and reflection, especially between the parents.

Data collection using interviews was influenced by Kvale and Brinkmann (Svend & Steinar, 2015) and was based on semi‐structured interview guides, which was divided into different research areas as “life as chronically ill,” “decision about respiratory assistance,” “having PCAs at home,” “consistency in the patient pathway” and “everyday life” based on a systematic literature search. The structure of the interview guide was identic for both children and parents about the research areas, but the levels of abstraction and reflection were different (Clark, 2010). Examples of questions for children were “Can you tell me about having PCAs around you?” and “Can you tell me about your everyday life?” but with a bit of adjustment about their age and illness, because it was not a homogeneous group. Examples of questions for parents were “How do you experience having PCAs at home?” and “How do you experience your everyday life and family life?”

On the basis of the families’ own choosing, the interviews were conducted at the hospital or their family home, and lasted from 46 to 98 min. All interviews were recorded on a smartphone and transcribed verbatim by the first author.

The Consolidated Criteria for Reporting Qualitative Research checklist was used as a guideline (Tong et al., 2007).

### 3.3 Sampling

Purposeful sampling was used when recruiting participants. To obtain data variety and sufficiency, we included participants with a chronic need for invasive or non‐invasive ventilation, either for some hours a day/night or 24h a day and having or having had PCAs at home; we also ensured variety in terms of age (0–18 years), gender, ethnicity, family patterns and new/experienced families and PCAs at home (Table 1). All children were diagnosed with chronic respiratory insufficiency and suffered from different diseases; neuromuscular diseases, lung diseases such as tracheomalacia and bronchopulmonary dysplasia, central apneas and facial anomalies.

### 3.4 Settings

The study was conducted at Respiratory Center West (RCW), Aarhus University Hospital, which is one of three centres in Denmark, which treats children <18 years with chronic respiratory insufficiency. The decision to offer respiratory treatment and surveillance is undertaken on the basis of shared‐decision‐making between the family and healthcare professionals such as chief physicians, nurses, social workers and financial assistants (Elwyn et al., 2012; Mesman et al., 2013). The Danish government and the Danish Health Authority support HMV for children to maintain their QOL and rehabilitation, and the treatment and surveillance are, therefore, financed by the child's home region and municipality. Families can receive financial help with, for example, rebuilding their home, transportation, supplies, respite care.

Families often get a team of six PCAs, who are requested from three cooperative care assistants’ recruitment agencies and are either nurses, social and healthcare assistants, social and healthcare helpers or personal helpers. All PCAs are trained and approved by nurses at RCW to provide surveillance and take care of the complex respiratory care and treatment such as ventilation, alarms, suctioning etc.

### 3.5 Data analysis

All textual data from the interviews were analysed by the first author and discussed by the research team using a method developed with inspiration from Ricoeur's theory (Dreyer & Pedersen, 2009; Ricoeur, 1973a). According to Ricoeur, text has to be interpreted, and following his thinking, “*what has to be interpreted in a text is what it says and what it speaks about*” (Ricoeur, 1973b) (p. 93). This method of analysis consists of three steps: naive reading, structural analysis and critical analysis and discussion (Dreyer & Pedersen, 2009). The naive reading is an initial interpretation of the whole text. In the structural analysis, the reader focuses on what the text says and speaks about, which, in this article, led to the development of four themes. Subsequently, discussion of the emerging themes created a deeper understanding of the interpretation (Dreyer & Pedersen, 2009). The research software NVivo 12.0 was used for data analysis.

### 3.6 Ethics

The study required no Research Ethics Committee approval from the National Committee on Health Research Ethics; however, it was approved by the legal office in the Central Denmark Region and adhered requirements from the Helsinki Declaration (World Med & World Medical, 2013). The participants were informed verbally and in writing about the aim, confidentiality, anonymization of identity, voluntary participation and the right to withdraw from the study at any time. All participants signed a consent letter and parents signed for their children. Interviewing children at different ages required several considerations about, for example, making them feel safe and creating an informal interview setting with short and concrete questions in an unprofessional language. All children had experienced being interviewed before.

## 4 RESULTS

The findings, drawing on naive understanding and structural analysis, are described in relation to four main themes.

### 4.1 Naïve understanding

Naïve understanding is the first step in a dialectical process between explaining and understanding, and it starts with an initial overall interpretation of the whole text and what it speaks about. Drawing, then, on the naïve understanding, families described how having HMV affected the family in several ways. Families explained how their everyday life, family life and social life were affected by HMV and how they felt limited in their behaviour and ability to express themselves. They articulated a mixture of emotions towards HMV, and parents in particular, had a deep desire and need to talk to someone about the situation. Parents struggled physically and mentally with practical things like ensuring the treatment of and care for their child and maintaining control. Families, parents and children, found it disabling, burdensome and sometimes frustrating to be entitled to PCAs: Yet, at the same time, they felt grateful for their help and the opportunity of HMV.

The next step in the analysis was to examine life with a child requiring HMV and PCAs seen from the family's perspective; this step offers interpretation based on the structural analysis.

### 4.2 Structural analysis

Structural analysis revealed four main themes: *“Forced to live an organized everyday life in dependency, ‘Family life was put to a challenging test’, ‘Coping with opposing emotions’* and *‘PCAs are indispensable but hard to integrate into family life’*.” Quotations are used to validate and unfold the interpretations.

### 4.3 Forced to live an organized everyday life in dependency

Living life as autonomously and independently as the child's condition permitted was what the parents wished for, however, having a child needing HMV treatment was time‐consuming and a huge responsibility, which for some families was hard to handle. It also necessitated practical help and support over periods of the day or 24h a day, which influenced the family's everyday life. The ill child on HMV became the family's focal point, and changed the family dynamic, where other family members had to adapt. *“There was a clear difference, yes, because we were always putting him in a bubble. His needs came first”* (8a). Living as a family with a child on HMV and PCAs was tantamount to having a different life compared to other families. Families sought for normalization; however, this was impossible “*It is difficult, because it means that our everyday lives are not as easy as others,” and it is dependent on his illness—we have to attend to his needs first”* (8a). On the other hand, families always tried to look at opportunities rather than limitations, and managed to find creative and alternative solutions to make everyday life more interconnected. *“We are probably a family who say as long as something has not been tested, we cannot say it does not work. We find a solution to things* (2a).”

Organizing the family's everyday life also had an impact on the interior of the house, because having a child on HMV required ventilator, respiratory devices, supplies, a room for PCAs and a wheelchair for those children who suffered from neuromuscular disease. Families had to move rooms and things around and experienced how the house easily turned into a hospital environment, unfortunately.

Some families also experienced challenges due to day care, nursery and school which affected them. Finding the right place where “they” could manage the child and PCAs and having the right support and willingness to understand and accommodate the child's illness and physical limitations were very important for the families. *“But there are some battles to be fought, because who wants him in day care and who wants a helper in the house?”* (11a). However, sending a respiratory ill child away from home to, for example, School or nursery was also transgressive, as families feared the risk of infection because pneumonia was life‐threatening for them. The many admissions and check‐ups at hospital also affected their everyday lives, as the child could not attend school and see their friends, parents had to take the time off work and siblings suffered deprivation.

Children on HMV attending school told how their friends always helped them to manage physical challenges. They experienced having good friendships, but they had to do other things, so play dates, sleepovers, parties and some sports were limited, which made them feel different. “*We play many fun games at school, and sometimes we use my wheelchair to spin around. But I cannot climb trees, which I really would like to try* (2b).”

### 4.4 Family life was put to a challenging test

Everyday life, social life and family life were affected for both parents and children on HMV and PCAs, and different from other families, because living as a family with a child on HMV became a family project. Social life often meant cancellation of many family events and social events or, for some families, hours of planning and packing. Some families really struggled with social and society barriers, but mostly, families accepted their situation with its limitations. *“We do not have PCAs* *from 4–8 p.m., so we have the opportunity there to just be us. A real family”* (2a) and *“We are often at home, but that's okay for me. I'm not using my wheelchair so much, only when my dad forces me to get out. I like being in my bed and gaming on my computer* (13b).”

Circumstances were even more complex when the parents’ relationship was affected, because having a child on HMV brought physical and psychological encumbrance into their partnership. They tried to handle it by finding time alone; however, having this common project also contributed to strengthening family life and some parents even felt a closer connection. *“In fact, I think we're stronger through it, actually. But, we are also bound to each other for good. Luckily, we manage to give each other space to do personal things* (12a).”

Work‐life balance was also an issue with mixed feelings about working. Some parents got loss of earnings made up by their municipality, but most parents worked full‐time/part‐time, and having flexibility in their jobs was essential for them because of the many hospital check‐ups/admissions and other disease‐related appointments.

*“If she has to go to the hospital or another appointment, one of us goes with her. Either we take the day off, without payment, or we try to compensate, and maybe work in the evening. We could not do it without our PCAs. It is really important for us to keep our jobs* (12a).”

However, common for the parents was the experience of working all the time, either at their jobs or at home, which was stressful.

Sharing resources between siblings and raising children differently proved to be challenging. Siblings’ lives were also affected because their needs came in second place, and the ill child on HMV became the family's focal point. Parents felt inadequate as they could not give their time and attention equally. *“Siblings are also forgotten in this—they should get a little more*… (8a).” Parents were aware of siblings’ needs, and some parents used different strategies to compensate and ensure a normal and free childhood, for example, to split the children up or to have time alone with the well sibling, allowing them to meet the well sibling's needs. *“Well, he also gets some experiences alone with us. We go mountain biking together, and he does lots of nature stuff with his mum. Each year we go for a weekend, a boys’ outing* (12a).”

The family house became the children's comfort zone, where only some of the children, HMV children and their siblings, invited friends over. Some children were embarrassed about the hospital environment in the house, but the reason for embarrassment was having PCAs at home. A mother told her well child's perspective: *“I cannot just play some strange games with my brother, because there is always someone watching him, and what I say and do* (8a).”

### 4.5 Coping with opposing emotions

Coping with opposing emotions meant having both positive and negative feelings towards the new situation with HMV and PCAs. On the one hand, families were very grateful for having been assigned respiratory treatment and surveillance and stated that they had a strong emotional attachment to their child and that they felt enriched at many levels. *“We are not very religious, but we believe that there is something called destiny* (8a).” On the other hand, families felt it was unfair, unexpected and extremely burdensome for the family.

“*I can remember when we just got her. I felt it was deeply unfair to me. Really. It was so unfair. I could just see all these challenges; almost like life crumbled away from me, because everything I thought I knew disappeared* *(…)* (12a).”

All of the families dealt with many thoughts and worries, *“Well, you can try to process it quietly. And as the years go by, it might be a bit easier, but the worry, I think, will always be there. Always* (13a).” Worries about treatment; seeing their child getting a tracheostomy was an especially painful and traumatic experience for parents and also the fact that their child's respiratory condition could change very quickly and the risk of a simple infection or pneumonia made them worry. Older children also raised concerns about treatment and their condition. *“I am deeply concerned about getting a fever. Sometimes, when I get a fever, I faint. I’m worried that I faint and will not wake up again* (6c).”

However, the new situation not only meant coping with opposing emotions; it also meant new daily routines, constant surveillance, high standards of hygiene, home cleanliness and many practical things that put parents under much pressure and were stressors, while they also made parents resilient. *“I just can't* *succumb to the pressure, and it is only me who has the overview and has to be like a project manager in all this* (7a).”

It was important for the families to have friends, family members, healthcare professionals or a psychologist to talk to, because not having someone to talk really affected the families negatively and made them feel lonely and vulnerable. *“When you* *(healthcare professionals)* *spend time talking to parents, we could feel it made a huge difference. Huge difference* (1a).*”* Networking with other families in a similar situation was very useful and was a great informal way to ask questions and discover that they were not the only ones. *“It really means a lot having a chat with someone who lives with it*. (10a).”

Navigating in health and social services systems became a significant stressor, because the systems were rigid and inflexible; and lack of communication, especially collaboration with their municipality, made families feel irritated, confused and sometimes even despondent. Parents dealt with fatigue and exhaustion, which they found to be increased when they had to deal with the different systems and administrations. However, families were dependent on help from public systems, which made them strive for overview and control. Many families used a basic family planner, whereas others chose to hire a private social worker, because they had a hard time understanding all the paragraphs in the legislation. *“There will always be some battles you have to fight. Just having a child who is sick is a struggle, and then you also have to fight against the system* (8a).”

### 4.6 PCAs are indispensable but hard to integrate into family life

Living in a family with a child with chronic respiratory illness and HMV meant being entitled to a team of PCAs, which brought mixed feelings to the family. On the one hand, families were grateful for having access to HMV, like it was a gift; but on the other hand, it was also hard and demanding. *“We are grateful, but the situation should have been different from start. It is not because we are not grateful. It's* *just because it is so unbelievably hard”* (5b) and *“I'm glad that I don't* *have a PCA anymore and can handle my mask myself* (9b).” Stability, cooperation and good communication in the teams were important, because the families were entitled to PCAs, either for some hours of the day/night or 24 hr, and because getting to know new PCAs was exhausting and time‐consuming. “*It was disabling for us having new PCAs all the time*” (3b) and “*We do not like our home being a railway station* (3a).” When new PCAs entered their home it was hard, and parents had bad conscience and could see their child behave differently. A child told “*Sometimes I shut down and deny to cooperate with new PCAs or temporary workers* (2b).”

*“It’s a big denial phase, because if you start thinking about how intrusive it is, then … I don’t* *think you could mentally take it at all. So, I pretend that it is just normal and is very nice. It is also sometimes, but it is not nice to be with other people 24 hours a day, whom you have not chosen yourself and you are never alone* (1a).”

When PCAs entered the house, it meant limited privacy for the family why the importance of having private spaces in the house where the PCAs were not allowed and also rules and agreements about when to have a formal and informal talk should be clear. *“We felt a bit like we were under surveillance, controlled in what to do and what to say and how to behave, but it got better over time* (8a).” Some parents said that they were being alienated in their own homes and they sometimes felt like they were under surveillance and not all families managed to relax and be themselves at home when PCAs were present.

An experience of one's home being invaded gave rise to a feeling of lack of control. Having control over the house, the child's care and treatment, practical and administrative aspects was essential for the families, especially the mothers. “*Suddenly, we could not decide over our own home, and that was hard. you had completely lost power over things you normally had before, and that was so strange* (5a).*”*

Just being able to be a parent and not being a caregiver all the time was important for parenthood, however, having PCAs made the division of roles clearer. *“Well, you have to remember being a mother in all this, because you share your child in some way* *(…)*. *And also make it clear for them that you are a PCA, you are not the mother to my child. I am* (10a).”

Most families preferred a professional relationship and collaboration with their PCAs based on trust, respect and clear agreements. However, few families had developed a personal relationship, where they not only talked about the HMW child, but also private matters and those families developed a kind of family tie which contributed to addressing the families’ lack of social life.

## 5 DISCUSSION

Living as a family with a child on HMV and dependent on PCAs was extremely challenging and burdensome. It became a family project involving all family members in several ways and, therefore, difficult to make family life work. Long‐term illness can result in a number of losses for almost all aspects of family life (Fay & Lesley, 2007; Israelsson‐Skogsberg et al., 2018).

Families were vulnerable and had mixed feelings towards the new situation, but a desire to live a normal life. Longing for normalization when living with long‐term illness has also been reported in other studies (Keilty & Daniels, 2018). Rehm and Bradley (2005), for example, claim that chronic conditions severely impact families’ way of living and organizing life, and how families do not fit into currently established attributes of normalization; however, families also recognize that a different life, also can be satisfying and enriching (Rehm & Bradley, 2005). Families were also able to achieve a sense of normality in their routines; nevertheless, home has become a public rather than a private sphere (Israelsson‐Skogsberg et al., 2018; Keilty & Daniels, 2018).

PCAs are indispensable, but integrating them into everyday life and family life were challenging and exhausting, and limited families’ privacy. Having little privacy at home and a feeling of social isolation has also been reported in other studies as being difficult for the family (Berit Lindahl & Lindblad, 2011b; Mah et al., 2008). Families had to make clear agreements with their PCAs about private space and alone time, however, being dependent on PCAs and public help was burdensome (Berit Lindahl & Lindblad, 2013). Parents feel responsible and involved in all aspects of their child's care and treatment and are overcome by tiredness and exhaustion, even though they have professionals around them (Heaton et al., 2005; B. Lindahl et al., 2011a).

Families in this study experienced opposing feelings, vulnerability and stress, similar to other findings on living as a family with a child on HMV or chronic disease in general. The psychosocial well‐being of children with chronic disease and their parents and siblings is affected (Barlow & Ellard, 2006). Families are at risk at developing internalizing and externalizing symptoms that affect the families’ QOL and physical and mental health (Barlow & Ellard, 2006; Caicedo, 2014). However, support from family members seems to mediate stress (Mah et al., 2008). In this study, sharing experiences with other families in similar situations and talking to professionals or someone closely related was very helpful and it contributes to families’ ability to cope with the situation. However, families are at risk at developing chronic sorrow, because long‐term illness can trigger a grief response in the child and family (Fay & Lesley, 2007).

### 5.1 Methodological reflections

A qualitative approach using method triangulation during data collection contributed to ensuring the study's validity. Families, parents and children's reflections and discussions provided long in‐depth narratives on lives lived with HMV and PCAs in a Scandinavian setting. It would have enriched the study if more children had been included; however, and, fortunately, the group of Danish children living on HMV is relatively small and we reached a point of data saturation, thus it was not a homogeneous group of children and families due to the sampling criteria. Besides, no sibling was included, only children and parents' perspective, which is a limitation, since the study explore family life.

Using the research software NVivo 12.0 strengthened the study because it helped to systematize the large amount of data. Subsequently, a Ricoeurian way of interpreting and constructing the data in three steps ensured transparency and in‐depth analysis, where four main themes were identified in the structural analysis. Credibility is reflected in an exhaustive analytical process undertaken by the first author with the second authors providing supervision and critical direction.

## 6 CONCLUSION

Being enduringly ill and having home‐based respiratory treatment differs from being cared for in hospital. Families are grateful for treatment at home; however, HMV and PCAs affect the family in several ways. Families suffer from mixed feelings and vulnerability. Families’ physical and emotional well‐being are affected; it is challenging and burdensome to make family life work when the family is dependent on public help for a lifetime. Living with PCAs affects the families’ privacy and they are hard to integrate PCAs into family life. HMV set‐ups seen from a professional and societal point of view need to be improved and supported differently, which requires preparation for family life with HMV and PCAs.

## 7 RELEVANCE FOR CLINICAL PRACTICE

Findings revealed new important knowledge about family life with HMV and PCAs and add to existing knowledge in this research area. Findings provide important directions for nursing care and clinical practice on how interventions, information, preparing and planning before and during life with HMV and PCAs should be more focussed, family‐centred and supportive in a long‐term perspective. However, further studies about children's perspectives on family life with HMV and PCAs are needed.

## CONFLICT OF INTEREST

The authors declare no conflicting interest about the research, authorship and/or publication of this article.

## Supporting information

Supplementary Material

## Data Availability

The data set used and analysed in the current study is available from the first author ABA on reasonable request and can be found in the computer system MidtX.
