# Supplementary material for: Living as a family with a child on home mechanical ventilation and personal care assistants—A burdensome impact on family life
Source: Nurs Open. 2021 May 5;8(6):3340–8. doi: 10.1002/nop2.879 (PMC8510762; doi:10.1002/nop2.879)
Supplement: Supplementary file 1 — Supplementary Material [file NOP2-8-3340-s001.docx]

# Supplementary File 1: COREQ checklist

Domain 1: Research team and reflexivity.

Personal Characteristics

1. Interviewer/facilitator Which author/s conducted the interview or focus group?

The first author did the individual and couple/family interviews.

1. Credentials What were the researcher’s credentials? E.g. PhD, MD

MScN, PhD (title page)

1. Occupation What was their occupation at the time of the study?

1. author: MscN, research nurse

2. author: MscN, Charge nurse

3. author: MD, EDIC

4. author: Professor, PhD, RN, MScN

(title page)

1. Gender Was the researcher male or female?

Female (title page)

1. Experience and training What experience or training did the researcher have?

The first author has experience from interviewing patients, relatives and health care professionals in Denmark and England and has experience during research, developing and quality in clinical practice during the last 10 years.

The last author is a senior researcher, professor in Denmark and Norway.

Relationship with participants

1. Relationship established Was a relationship established prior to study commencement?

No, not for the 1. author.

1. Participant knowledge of the Interviewer What did the participants know about the researcher? e.g. personal goals, reasons for doing the Research

None

1. Interviewer characteristics What characteristics were reported about the interviewer/facilitator? e.g. Bias, assumptions, reasons and interests in the research topic

None

Domain 2: study design (see page 3-6 in the main manuscript).

Theoretical framework

1. Methodological orientation and Theory What methodological orientation was stated to underpin the study? e.g. grounded theory, discourse analysis, ethnography, phenomenology, content analysis, participant selection

A phenomenological hermeneutic approach (page 3-4, 6)

1. Sampling How were participants selected? e.g. purposive, convenience, consecutive, snowball

Purposeful sampling (page 4-5)

1. Method of approach How were participants approached? e.g. face-to-face, telephone, mail, email

Face-to-face (page 3-4)

1. Sample size How many participants were in the study?

22 (page 3 and Table 1)

1. Non-participation How many people refused to participate or dropped out? Reasons? Setting

None

1. Setting of data collection Where was the data collected? e.g. home, clinic, workplace

At Respiratory Center west or their family house (page 4-5)

1. Presence of non-participants Was anyone else present besides the participants and researchers?

Parents when interviewing the children except the child under family 13 (Table 1)

1. Description of sample What are the important characteristics of the sample? e.g. demographic data, date, Data collection

Parents, ill children with HMV (page 4-5 and Table 1)

1. Interview guide Were questions, prompts, guides provided by the authors? Was it pilot tested?

Provided by the authors (page 4)

1. Repeat interviews Were repeat interviews carried out? If yes, how many?

None

1. Audio/visual recording Did the research use audio or visual recording to collect the data?

Audio (page 4)

1. Field notes Were field notes made during and/or after the interview or focus group?

No

1. Duration What was the duration of the interviews or focus group?

Average 45 min (46 minutes to 98 minutes) (page 4)

1. Data saturation Was data saturation discussed?

Yes (page 4 and 15)

1. Transcripts returned Were transcripts returned to participants for comment and/or correction?

No

Domain 3: analysis and findings

Data analysis

1. Number of data coders How many data coders coded the data?

39

1. Description of the coding tree Did authors provide a description of the coding tree?

No

1. Derivation of themes Were themes identified in advance or derived from the data?

No

1. Software What software, if applicable, was used to manage the data?

NVivo 12

1. Participant checking Did participants provide feedback on the findings?

No

Reporting

1. Quotations presented Were participant quotations presented to illustrate the themes / findings? Was each quotation identified? e.g. participant number

Yes (page 8-13)

1. Data and findings consistent Was there consistency between the data presented and the findings?

Yes

1. Clarity of major themes Were major themes clearly presented in the findings?

Yes (page 7-14)

1. Clarity of minor themes Is there a description of diverse cases or discussion of minor themes?

Yes discussion of the themes (page 14-15)
